# Giant Hemangiomatous Adrenal Cyst Mimicking a Hemorrhagic Hepatic Cyst: A Case Report

**DOI:** 10.70352/scrj.cr.26-0312

**Published:** 2026-07-02

**Authors:** Yuna Saito, Naoko Fukushima, Kazuto Tsuboi, Masatoshi Arai, Hiroaki Kitamura, Seiya Yoshida, Tetsuya Kajimoto

**Affiliations:** 1 Department of Surgery, Fuji General Hospital, Fuji, Shizuoka, Japan; 2 Department of Surgery, The Jikei University School of Medicine, Tokyo, Japan

**Keywords:** adrenal cyst, hemangiomatous cyst, hepatic cyst, vascular type, hemorrhage, differential diagnosis

## Abstract

**INTRODUCTION:**

Adrenal hemangiomatous cysts are rare. Giant cysts of the right adrenal gland may be difficult to distinguish from hepatic cysts owing to their close anatomical proximity to the liver. Here, we report a case of a hemangiomatous adrenal cyst mimicking a hemorrhagic hepatic cyst.

**CASE PRESENTATION:**

A 48-year-old woman presented with a 10-day history of abdominal pain and distention. The laboratory findings revealed marked inflammation and severe anemia. Contrast-enhanced CT demonstrated a 28-cm cystic lesion extending from the right hepatic lobe, with peripheral calcification and intralesional high-attenuation areas suggestive of a hemorrhage. A hemorrhagic hepatic cyst was initially suspected and percutaneous drainage was performed. However, the drained fluid became increasingly hemorrhagic, and anemia progressed despite repeated transfusions, indicating ongoing intracystic bleeding; therefore, exploratory laparotomy was undertaken. En bloc resection of the cyst, hepatic segment VI, and the right adrenal gland was performed. Histopathological examination revealed dilated vascular channels lined with endothelial cells that were CD34-positive and D2-40-negative and were contiguous with the adrenal cortex, confirming the diagnosis of a hemangiomatous adrenal cyst. The postoperative course was uneventful, and no recurrence was observed at 6-month follow-up.

**CONCLUSIONS:**

Here, we report a rare case of a giant hemangiomatous adrenal cyst. Because large right adrenal cysts may be difficult to distinguish from hepatic cysts owing to their anatomical proximity, adrenal cysts should be considered in the differential diagnosis of large upper abdominal cystic lesions.

## Abbreviations

Hb
: hemoglobin
IVR
: interventional radiology

## INTRODUCTION

Adrenal cysts are rare, found in 0.06% of anatomical specimens, with the majority being nonfunctional and asymptomatic. Among these, hemangiomatous cysts are an uncommon subtype accounting for only 3% of all adrenal cysts.^1)^ These are characterized by cyst walls lined with CD34-positive endothelial cells.^2)^ Most adrenal cysts are small and incidentally detected, whereas giant adrenal cysts are extremely rare.^3)^ In particular, giant right adrenal cysts may be difficult to distinguish from hepatic cysts arising from the caudate lobe or posterior segment due to their anatomical proximity to the liver, making preoperative diagnosis challenging. We report a case of a giant hemangiomatous adrenal cyst that mimicked a hemorrhagic hepatic cyst.

## CASE PRESENTATION

A 48-year-old woman with no remarkable medical history presented with a 10-day history of progressive abdominal pain and distention. On physical examination, her vital signs were stable and abdominal distention with spontaneous pain and localized tenderness on the right side of the abdomen were noted; however, no obvious signs of peritoneal irritation were observed. Laboratory tests showed significant inflammatory findings, with a white blood cell count of 12100/μL, and C-reactive protein level of 15.62 mg/dL. Severe anemia was also noted, with an Hb concentration of 5.6 g/dL, whereas the other laboratory parameters were within normal ranges. Contrast-enhanced CT revealed a large cystic lesion measuring approximately 28.5 cm in maximum diameter, extending caudally from the right hepatic lobe, accompanied by peripheral calcification (**Fig. 1A**). The high-attenuation area within the cyst suggested intralesional hematoma formation, although no active contrast extravasation was observed (**Fig. 1B**). The right adrenal gland could not be clearly identified. The right kidney and inferior vena cava were markedly compressed to the left by the cyst, with both structures exhibiting well-defined borders (**Fig. 1C**). Ultrasonography revealed a large cystic lesion corresponding to the lesion identified on CT. Based on these findings, a hemorrhage and infection of the hepatic cyst were strongly suspected. US-guided cyst aspiration and drainage were performed on the day of admission. The initial drainage fluid was pale bloody (Hb 0.5 g/dL); however, the drainage fluid gradually became more hemorrhagic (Hb 2.3 g/dL). Repeated CT scans showed no evidence of active extravasation. Because of severe anemia on admission, she received a transfusion of 8 units of packed red blood cells until the 2nd hospital day, resulting in an improvement in Hb level to 9.3 g/dL. Despite this temporary improvement, the Hb level declined to 7.7 g/dL on the 3rd hospital day, suggesting ongoing bleeding. A temporary reduction in the drainage output suggested catheter obstruction due to clot formation. After replacement with a larger-diameter drainage tube, an additional 3000 mL of bloody fluid (Hb 5.3 g/dL) was obtained. Despite repeated dynamic CT examinations, the source of bleeding remained unidentified. As hemostasis by IVR was considered unfeasible, exploratory laparotomy was performed to control the ongoing hemorrhage. During laparotomy, hemorrhagic ascites were observed, and the cyst partially collapsed. Upon incision of the cyst, a large intraluminal hematoma weighing approximately 1000 g was identified along with bloody cystic fluid (Hb 5.0 g/dL) (**Fig. 2A** and **2B**). Although the hematoma and the cystic fluid were removed, no active bleeding sites were identified. Therefore, complete surgical resection was necessary to achieve definitive hemostasis. The cyst was firmly adherent to hepatic segment VI (**Fig. 2C**) and the right adrenal gland (**Fig. 2D**); therefore, en bloc resection of the cyst, hepatic segment VI, and right adrenal gland was performed (**Fig. 3**). The operation time was 189 min, with an intraoperative blood loss of 1480 g. Histopathological examination revealed that the cyst wall was in direct contact with the thin adrenal cortex (**Fig. 4A**). The cyst wall was composed of markedly dilated vascular channels lined with flattened endothelial cells that were positive for CD34 and negative for D2-40 (**Fig. 4B**–**4D**). These findings confirmed the diagnosis of adrenal hemangiomatous endothelial cyst. Postoperatively, although a transfusion of 4 units of packed red blood cells was required, the Hb level gradually improved. The patient was discharged on POD 15 without any complications. No recurrence was observed during the 6-month follow-up period.

**Fig. 1:**
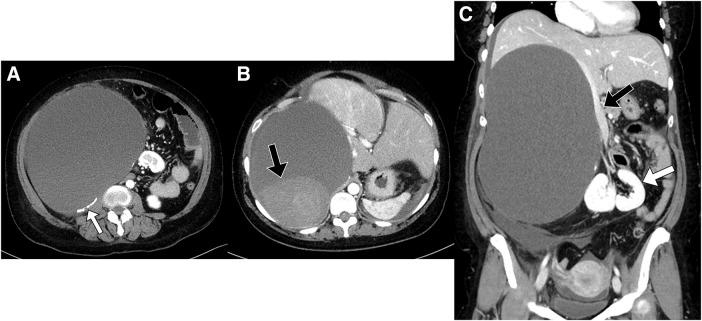
Enhanced abdominal CT findings. (**A**, **B**) Enhanced abdominal CT showed a cyst with peripheral calcification (white arrow), and the high-attenuation area within the cyst (black arrow). (**C**) The right kidney (white arrow) and inferior vena cava (black arrow) are markedly displaced to the left by the cyst, with well-defined borders.

**Fig. 2:**
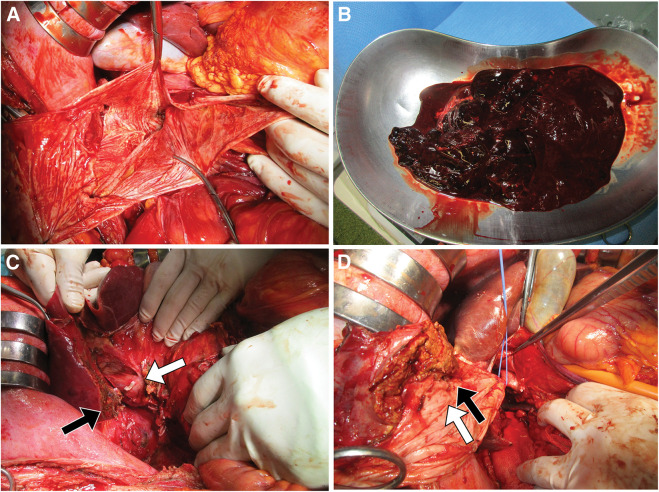
Intraoperative findings. (**A**) The cyst was opened and drained, and the intracystic fluid and hematoma were evacuated. (**B**) Hematoma retained within the cyst cavity. (**C**) The cyst was adherent to the inferior vena cava (white arrow) and the hepatic segment VI. Partial resection of hepatic segment VI was performed (black arrow). (**D**) The cyst was firmly adherent to the right adrenal gland (white arrow). During the dissection of the right adrenal gland from the surrounding tissues, the right adrenal vein was identified (black arrow).

**Fig. 3:**
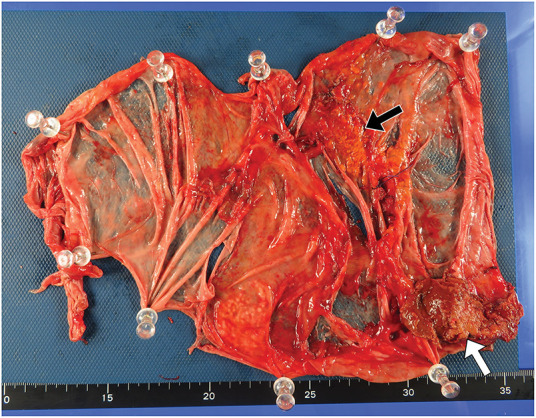
Resected specimen. The cyst, hepatic segment VI (white arrow), and the right adrenal gland (black arrow) were resected en bloc.

**Fig. 4:**
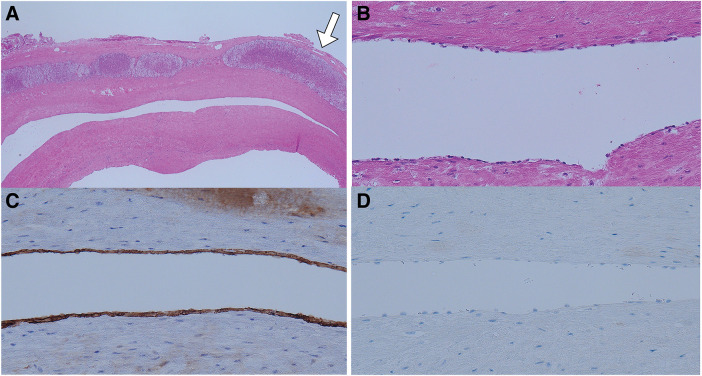
Histopathological findings. Histopathological examination showed the cyst wall in direct contact with the thin adrenal cortex (white arrow; **A**) (×2), and was composed of markedly dilated vascular channels lined by flattened endothelial-like cells (**B**) (×20). Immunohistochemical staining demonstrated that these lining cells were positive for CD34 (**C**) and negative for D2-40 (**D**) (×20).

## DISCUSSION

Adrenal cysts are rare lesions. Foster^3)^ classified adrenal cysts as parasitic, epithelial, endothelial, or pseudocysts. Endothelial cysts are further subdivided into lymphangiomatous and hemangiomatous types, the latter representing an extremely rare subtype of adrenal cysts. Most adrenal cysts are <10 cm, typically unilateral, and occur predominantly in women in the 4th to 5th decades of life.^3)^ Previous studies have suggested that adrenal tumors, regardless of benign or malignant pathology, may occur more frequently in women because of hormonal interactions involving adrenal and endocrine functions.^4)^ In addition, estrogen has been reported to promote endothelial-dependent vasodilation, angiogenesis, and endothelial healing, which may partially contribute to the female predominance of endothelial cysts.^5)^ In the present case, a 28-cm hemangiomatous cyst arising from the right adrenal gland in a woman in her 40s represented an exceptionally large and rare presentation.

Endothelial adrenal cysts are characterized by a wall lined with endothelial cells.^6)^ Immunohistochemistry aids in subtype differentiation; lymphangiomatous cysts are positive for D2-40, whereas hemangiomatous cysts are positive for vascular markers, such as CD31, CD34, and factor VIII-related antigen.^2,6)^ In this case, the presence of CD34-positive and D2-40-negative endothelial linings supported the diagnosis. Although their precise pathogenesis remains unclear, these lesions are thought to arise from the dilatation of adrenal capillaries or congenital vascular malformations.^7)^

Management depends on the symptoms, hormonal activity, imaging findings, and the possibility of malignancy. While asymptomatic cysts with benign features may be observed, lesions that are symptomatic, show interval growth, or are diagnostically uncertain generally require surgical resection. Although minimally invasive adrenalectomy is widely performed for benign tumors, giant cysts or cases complicated by active hemorrhage may necessitate open surgery.^8,9)^ Percutaneous drainage has been reported for selected benign cysts^10)^; however, caution is required for vascular lesions. In hemangiomatous cysts, decompression may alter the intracystic pressure dynamics and potentially precipitate or exacerbate bleeding. In the present case, we performed drainage for diagnostic and palliative purposes, because we suspected a hemorrhagic hepatic cyst based on the imaging findings. In retrospect, additional imaging modalities such as MRI or short-interval radiological follow-up before drainage might have been preferable to better characterize the lesion and determine the organ of origin. As the lesion was considered to be a benign hepatic cyst, we thought that surgery would not be necessary if the symptoms improved. However, the Hb concentration of the drained fluid progressively approached that of the peripheral blood, and anemia worsened despite repeated transfusions, suggesting persistent intracystic hemorrhage. Ultimately, exploratory laparotomy was chosen for safe en bloc resection and definitive hemostasis. Since no active bleeding sites were identified during the operation, and it was also uncertain whether the cyst was of hepatic or adrenal origin, en bloc resection, including partial hepatectomy and adrenalectomy, was performed for diagnostic treatment purposes. Consequently, the procedure became more invasive. If the correct diagnosis and adrenal origin had been recognized preoperatively, elective surgery without prior drainage might have been feasible and could potentially have avoided emergency surgery and excessive surgical invasiveness.

The right adrenal gland lies in the retroperitoneum, adjacent to the posterior hepatic segments. In giant adrenal cysts, progressive enlargement may obscure the fat plane and compress the liver, making the identification of the organ of origin challenging. Radiological signs, such as the beak (claw) sign or the embedded organ sign, may help determine the site of origin^11)^; however, these findings are not specific and may be absent when the adrenal parenchyma becomes markedly thinned. In the present case, none of these features was demonstrable. The 28-cm cyst compressed the hepatic segment VI and contained high-attenuation areas suggestive of hemorrhage, closely resembling a hemorrhagic hepatic cyst. Consequently, the preoperative determination of the adrenal origin was difficult. Previous reports have described the diagnostic difficulty in distinguishing large adrenal cysts from hepatic cystic lesions.^12)^ Therefore, adrenal cysts should be considered in the differential diagnosis of large cystic masses that lack definitive hepatic features.

A PubMed search using the keywords “vascular adrenal cyst,” “hemangiomatous adrenal cyst,” and “angiomatous adrenal cyst” identified 9 previously reported cases of hemangiomatous adrenal cysts (**Table 1**).^13–21)^ Including our case, a total of 10 cases were analyzed. The reported age range was broad, ranging from 18 to 76 years, and all but 1 patient was female. Four cases arose in the right adrenal gland and 6 in the left adrenal gland. CT findings were variable, including differences in locularity, internal homogeneity, calcification, and mass effect. MRI was performed in only 5 cases, and the findings were also variable, with differences in signal intensity patterns. Five patients presented with abdominal symptoms, and 3 of them showed intracystic bleeding. However, intracystic bleeding was also found in asymptomatic patients or in those with non-abdominal symptoms. The remaining 5 cases were incidentally detected during the evaluation for nonspecific symptoms and subsequently underwent surgical resection due to suspected malignancy or evidence of interval growth. The interval from symptom onset to surgery ranged from 1 month to 2 years, whereas the interval in the present case was exceptionally short at only 3 days. Most lesions measured approximately 5 cm in diameter and only 3 cases involved giant cysts exceeding 20 cm, highlighting their extreme rarity. To the best of our knowledge, this is the largest reported hemangiomatous adrenal cyst arising from the right adrenal gland.

**Table 1:** Reported cases of adrenal hemangiomatous cysts

Author	Year	Age	Sex	Symptoms	Size (cm)	Location	Imaging feature (CT)	Imaging feature (MRI T1WI/T2WI)	Symptom onset to surgery	Properties of the cyst contents	Surgery
Sell et al.^13)^	1994	26	F	Abdominal pain, weight loss	23 × 21	Right	Multilocular, mass effect	—	Uncertain	Blood	Uncertain
Hirota et al. ^14)^	1999	55	F	Abdominal pain, headache	4 × 3	Left	Unilocular, inhomogeneity, calcification	High and low/ high and low intensity	2 months	Blood, calcification	Uncertain
Narayana et al.^15)^	2010	18	F	Abdominal pain	9.1 × 6.8	Right	Unilocular, mass effect	—	1 month	—	L-adrenalectomy
Yamada et al.^16)^	2011	72	F	None	3.7 × 2.7 × 2.5	Left	Unilocular, inhomogeneity	—	Uncertain	Nodules	Adrenalectomy
Morse et al.^17)^	2014	36	F	Headache, palpitation, hypertension	4.4 × 2.5 × 1.5	Right	Unilocular, homogeneity	—	Uncertain	—	L-adrenalectomy
Sakaue et al.^18)^	2021	67	F	None	5.2 × 5.0	Left	Unilocular, inhomogeneity	High/low intensity	Uncertain	Blood, nodules	L-adrenalectomy
Wu et al.^19)^	2022	76	M	None	6.4 × 4.7 × 3.9	Left	Unilocular, homogeneity	High and low intensity/none	Uncertain	Blood, calcification	L-adrenalectomy
Yu et al.^20)^	2023	38	F	Abdominal distention	31 × 17 × 16	Left	Unilocular, homogeneity. calcification, mass effect	High/low intensity	1 year	—	Adrenalectomy
Asai et al.^21)^	2025	56	F	Hypertension	5.2	Left	Multilocular, homogeneity, calcification	High/low intensity	2 years	Blood	R-adrenalectomy
Our case	2025	48	F	Abdominal pain, distention	28.5 × 20.3 × 16.0	Right	Unilocular, homogeneity, calcification, mass effect	—	3 days	Blood	Adrenalectomy

F, female; L, laparoscopic; M, male; R, robotic

## CONCLUSIONS

Here, we report a rare case of a hemangiomatous adrenal cyst treated with surgical resection. When large, these lesions may mimic hepatic cysts. Therefore, adrenal cysts should be considered in the differential diagnosis of large cystic lesions.

## DECLARATIONS

### Availability of data and materials

The data that support the findings of this study are available from the corresponding author upon reasonable request.
